# On the Potential Usefulness of Fourier Spectra of Delayed Fluorescence from Plants

**DOI:** 10.3390/s141223620

**Published:** 2014-12-09

**Authors:** Ya Guo, Jinglu Tan

**Affiliations:** Department of Bioengineering, University of Missouri, Columbia, MO 65211, USA; E-Mail: tanj@missouri.edu

**Keywords:** Fourier transform, delayed fluorescence, photosynthesis, PSII

## Abstract

Delayed fluorescence (DF) from photosystem II (PSII) of plants can be potentially used as a biosensor for the detection of plant physiological status and environmental changes. It has been analyzed mainly in the time domain. Frequency-domain analysis through Fourier transform allows viewing a signal from another angle, but the usefulness of DF spectra has not been well studied. In this work, experiments were conducted to show the differences and similarities in DF spectra of different plants with short pulse excitation. The DF spectra show low-pass characteristics with first-order attenuation of high frequencies. The results also show that the low-frequency components differ while the high-frequency components are similar. These may imply the potential usefulness of Fourier spectra of DF to analyze photoelectron transport in plants and classify samples.

## 1. Introduction

In photosynthesis, a photon can excite an electron of a chlorophyll molecule in photosystem II (PSII) to a higher energy level. When the higher-energy-level electron returns the ground state, a new photon is produced. This new photon is the commonly called prompt chlorophyll *a* fluorescence (PF) of PSII [1–4]. Its lifetime is usually longer (in the order of pico- or nanoseconds). The excited electron can also be transferred along the electron transport chain of PSII and finally be used for photochemical reactions. Chemical reactions are usually reversible; therefore, the electron can be potentially transferred back. When it is transferred back, it may result in a chlorophyll *a* molecule (e.g., P680 or PSII antenna chlorophyll molecules) in the excited state. This excited chlorophyll molecule is also capable of emitting chlorophyll fluorescence. It takes time for the electrons on the electron transport chain of PSII to transfer back to produce chlorophyll fluorescence. This type of chlorophyll *a* fluorescence is thus commonly referred to as delayed fluorescence (DF, also known as delayed light, delayed luminescence or DL) because it has a much longer lifetime (minutes or even hours) [5]. In this work, DF refers to delayed chlorophyll *a* fluorescence from PSII of plant leaves for conciseness. The emission of DF from PSII of plant leaves represents a backflow of energy captured for photosynthetic activities [6–8]. Anything that affects photosynthesis can be potentially reflected in DF emitted from PSII [9–11]. It is thus DF can be potentially used as a universal biosensor to sense plant physiological status and environmental changes. The usefulness of DF from PSII for various applications has been summarized by Guo and Tan [12], which include assessment or detection of photosynthesis rate, plant circadian, plant senescence, nutrients, salt stress, chilling stress, heat stress, drought stress, acid rain, herbicide, metal pollution, and aquatic ecosystems. An attempt to analyze DF in the frequency domain can be found in Guo and Tan [13]; however, DF from PSII has been mainly analyzed in the time domain [12,14–16].

Analyzing a signal in the frequency domain through Fourier transform (FT) allows viewing the signal from a different angle [17–20]. The usefulness of DF spectra in DF analysis, however, has not been well studied. It is not clear what differences and similarities may exist in the DF spectra of different plants, and it is not known whether the DF spectra are useful in revealing the PSII kinetic behavior as affected by chemical and other stresses and in sensing plant physiological status and environmental changes.

In this work, we show whether DF spectra are useful in indicating some basic kinetic characteristics of photo-electron transduction in plants and the effect of a chemical stress. Experiments were conducted to measure and compare the DF spectra of different plants or of the same plant but at varied levels of herbicide stress. Samples used included both trees and vegetables. Deserved to mention, DF emission is light intensity and duration dependent. While the electron transport activities of PSII can be modeled as linear model structure with short pulse excitation [21], strong light will trigger complex nonlinear processes in the electron transport system. The complexities induced by strong light bring a lot of difficulty to interpret and compare DF from different samples. On another side, the complex nonlinear processes are not necessary for comparison between samples. For simplification, this work focuses on the DF excited by short pulse, which is not sufficient to saturate the system. The excitation light is the same for all the samples, which makes the DF spectra comparable between samples.

## 2. Measurements

To test the usefulness of Fourier spectra of DF, DF from different varieties of plants or the same variety of plants but with different levels of chemical stress was measured, including two tree species, three vegetable species, and garden bean leaves with three levels of herbicide stress. For the two tree species, DF from magnolia and *Schefflera arboricola* was measured. The magnolia tree grew naturally on the campus of the University of Missouri. The *Schefflera* plants were grown in a hallway with large windows. Sunlight could shine on the plants in the afternoon. Regular irrigation was applied to the *Schefflera* plants. The environmental temperature of the hallway was 25 °C.

For the vegetable species, leaves of lettuce, spinach, and garden bean plants were used. The garden bean plants were grown in pots (10-cm height and 9-cm diameter) indoors under fluorescent lighting (12:12-h on-off cycle) and in constant room temperature of 25 °C. Water was regularly applied to irrigate the plants. The lettuce and spinach plants were obtained from a local store. Because the effect and mechanism of 3-(3,4-dichlorophenyl)-1,1-dimethylurea (DCMU) on plant photosynthesis is well known, DCMU was used as the test chemical stress in this research. Garden bean leaves were submerged in an 80-μM DCMU solution for 0, 15, and 60 min to give varied chemical stress levels.

A red LED with peak emission at 680 nm (L680-06AU, Marubeni, Santa Clara, CA, USA) was used to excite the samples. The excitation light was delivered through an 8-mm liquid light guide (77628, Oriel, Irvine, CA, USA). The DF photons were fed into a channel multiplier tube (CMT, MH1373P, PerkinElmer,Waltham, MA, USA) through another liquid guide. The output pulses of CMT were recorded with a gated photon counter/multiscaler card (PMS-400, Becker & Hickl GmbH, Berlin, Germany). The sampling frequency was set at 100 Hz and the total sampling time was 2 min. A diagram and detailed explanation of the experimental setup can be found in Guo and Tan [22]. For each species or stress level, five samples were measured. Samples were dark-adapted 30 min and excited with a 0.5-s illumination pulse. The illumination intensity and pulse width were experimentally determined so that they did not saturate the system.

## 3. Results

### 3.1. DF Spectra of Tree Samples

Differences in samples such as age and orientation may affect the intensity of DF. Since the overall DF intensity does not affect shape of the DF spectrum, each DF response was normalized by its maximum value before further analysis.

**Figure 1.**
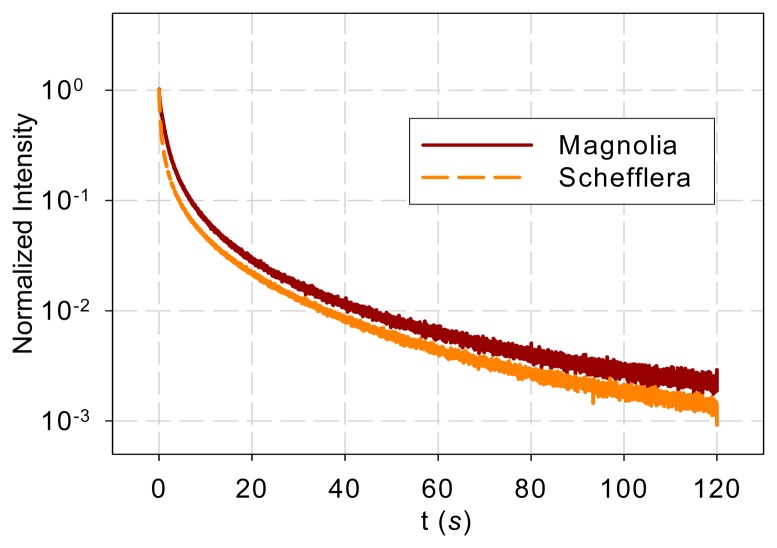
DF from magnolia and *Schefflera* leaves following the 0.5-s excitation light in the time domain.

**Figure 2.**
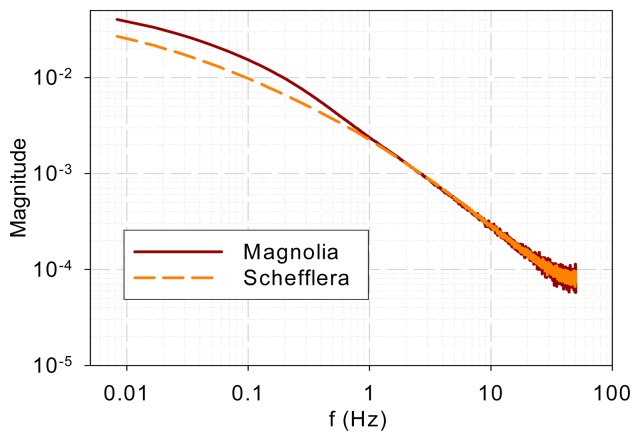
DF spectra for magnolia and *Schefflera* samples.

Figure 1 shows the normalized DF from magnolia and *Schefflera* samples in the time domain and Figure 2 shows the DF spectra of the two tree species. Figure 2 reveals that the DF frequency characteristics of the two tree species differ mainly in the low-frequency range (<1 Hz). The curves overlap at high frequencies (>1 Hz).

**Figure 3.**
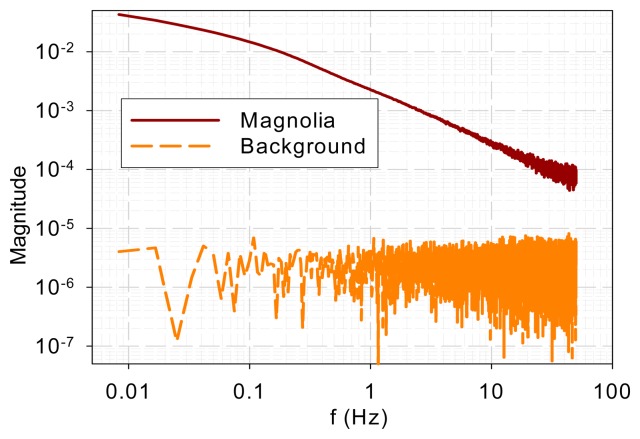
Comparison of DF and background spectra.

To ensure that the similarity in high-frequency characteristics was not due to common background noise, background noise was measured when a sample was present. The magnolia DF and background spectra are shown in Figure 3. It is evident that the spectra bear little resemblance. The sample DF shows typical low-pass characteristics while the background signal gives a flat spectrum, which indicates a white noise.

### 3.2. DF Spectra of Vegetable Samples

**Figure 4.**
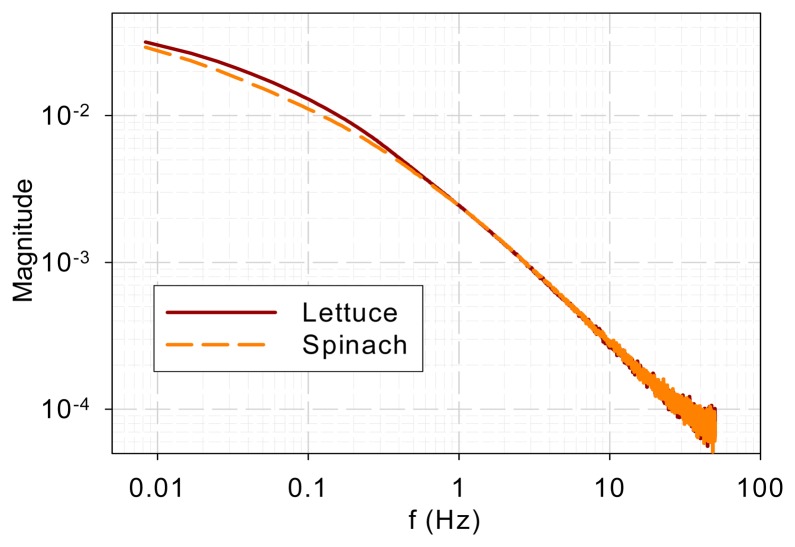
DF spectra of lettuce and spinach samples.

Figure 4 shows the DF spectra of lettuce and spinach samples. Similar to the two tree species, the two spectra show low-pass characteristics and they are different in the low-frequency range (<0.3 Hz) but similar in the high-frequency range (>0.3 Hz).

### 3.3. Herbicide Effect on DF Spectra

**Figure 5.**
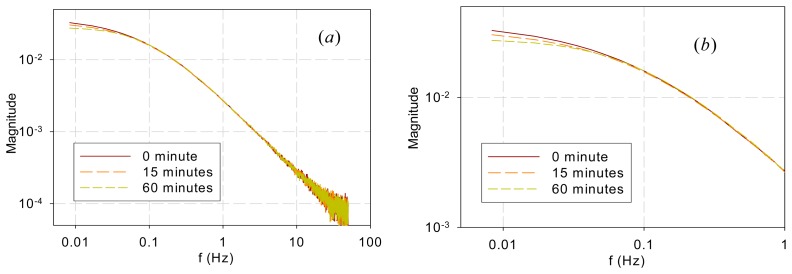
DF spectra of garden bean leaves at different herbicide binding times (**a**) Full computed spectra; (**b**) Zoom-in to show differences.

Figure 5 shows the DF spectra of garden bean leaves after they were submerged in an 80-μM DCMU solution for 0, 15, and 60 min. Again, the spectra are similar in the high-frequency range (>0.04 Hz) but different in the low-frequency range (<0.04 Hz). It is interesting to note that the bifurcating frequency of 0.04 Hz is much lower than that (1 Hz) for the two trees and that (0.3 Hz) for the vegetables.

## 4. Discussion

**Figure 6.**
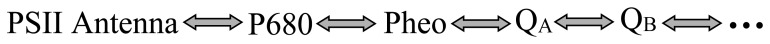
Early stages of the PSII photoelectron transport chain.

In photosynthesis, a photon captured by PSII antennas may excite a chlorophyll molecule (Chl or P680) and carried forward for photochemical reactions by an electron transport chain of pheophytin (Pheo), plastoquinone A (Q_A_), plastoquinone B (Q_B_), and subsequent steps as depicted in Figure 6. There is a chance of reverse reaction for each step [23,24] and DF emission results from reverse reactions [6–8]. As such, the DF spectra are a manifestation of the photochemical reaction kinetics involved in DF emission.

The reaction kinetics can be modeled at different levels of complexity. By lumping the fast reactions involving P680 and Pheo, Guo *et al.*, modeled the early stages of the photoelectron transport kinetics with a 5th-order linear model as follows when excitation light is not so strong that saturates the system [21].

(1) $$\left\{\begin{array}{c} {ẋ}_{1}\\ {ẋ}_{2}\\ {ẋ}_{3}\\ {ẋ}_{4}\\ {ẋ}_{5} \end{array}\right\}=\text{A}\;\left\{\begin{array}{c} x_{1}\\ x_{2}\\ x_{3}\\ x_{4}\\ x_{5} \end{array}\right\}+\left\{\begin{array}{c} k_{1}\\ 0\\ 0\\ 0\\ 0 \end{array}\right\}u$$

with:

(2) $$\text{A}=-\left[\begin{array}{ccccc} \left(k_{1}u+k_{2}+k_{3}\right) & \left(k_{1}u-k_{4}\right) & k_{1}u & k_{1}u & \left(k_{1}u-k_{5}\right)\\ -k_{3} & \left(k_{1}u+k_{4}\right) & -k_{2} & 0 & 0\\ 0 & -k_{1}u & \left(k_{2}+k_{3}\right) & -k_{4} & 0\\ 0 & 0 & -k_{3} & \left(k_{1}u+k_{4}+k_{5}\right) & -k_{2}\\ 0 & 0 & 0 & -k_{1}u & \left(k_{2}+k_{5}\right) \end{array}\right]$$

where *x*_1_ through *x*_5_ are state variables representing the redox status combinations of Q_A_ and Q_B_, A is the system matrix, *k*_1_ through *k*_5_ are reaction rate constants, and *u* is the intensity of illumination. DF emission is given by:

(3) $$DF=k_{6}\;k_{2}\;(x_{1}+x_{3}+x_{5})$$

where *k*_6_ is an overall gain factor.

DF is only practically measureable after the excitation light is turned off; thus measured DF as shown in Figure 1 is an initial condition response and the initial conditions are the values of the state variables at the end of illumination ([*x*_10_, *x*_20_, …, *x_n_*_0_]^T^). For a pulse illumination of constant intensity *u*, DF can be expressed as [13,25,26]

(4) $$DF=\left[\phi_{1},\phi_{2},\cdots ,\phi_{n}\right]\;\left[\begin{array}{l} e^{\lambda_{1}t}\\ e^{\lambda_{2}t}\\ \vdots \\ e^{\lambda_{n}t} \end{array}\right],(n=5)$$

where [ϕ_1_, ϕ_2_, …, ϕ*_n_*] is a constant vector and [λ_1_, λ_2_, …, λ*_n_*] is the eigenvalue vector of A. For a given constant *u*, the two vectors are entirely determined by the *k* values (*k*_1_ through *k*_6_). After optimization of the *k* values, the model was found capable of capturing the kinetic behaviors in measured DF under varied conditions [21].

Fourier transform of Equation (4) gives the DF spectrum as:

(5) $$S_{DF}(f)=\left|\sum_{j=1}^{n}\phi_{j}\;\frac{1}{-\lambda_{j}+i2\pi f}\right|$$

where $i=\sqrt{-1}$ is the imaginary unit.

Equation (5) shows that the DF spectrum can be expressed as a weighted sum of polynomial fractions in *f*. While this is not surprising as Equation (5) is generally true for any linear system, the expression allows several points to be made from the measured DF spectra presented earlier.

**Figure 7.**
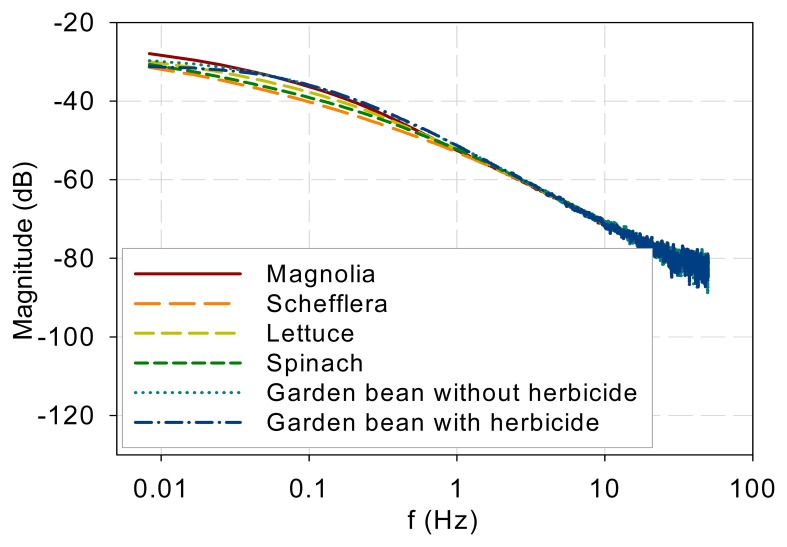
DF spectra of all the measured tree and vegetable plants.

- The measurement results indicate that the DF spectra for different plants and DCMU levels have similar low-pass characteristics and differ only at low frequencies. Figure 7 plots all the measured DF spectra together and shows the convergence of the spectra beyond approximately 1 Hz. The eigenvalues (λ*_j_*, *j* = 1, 2, …, *n*) in Equation (5) indicate the frequency at which each pole or mode begins to influence the shape of the spectrum. The spectral convergence thus means that the eigenvalues involved in shaping the DF kinetics are less than 1 Hz based on the measurements made. Although the 1 Hz value may not be general and more experiments are needed, the low-pass nature plus an eigenvalue upper bound supports Guo and Tan [16] and Guo *et al.* [21] in that the early stages of the PSII electron transport chain can be modeled with low-order models. Additionally, a measurement frequency greater than 50–100 Hz is not necessary to capture the system kinetics.
- The spectra beyond 1 Hz in Figure 7 are straight lines descending at 20 dB per decade. This means that when the fractions in Equation (5) are summed up, the order of the denominator polynomial is higher than that of the numerator by one. This indicates that though there are variations and differences in the low-frequency behavior, the high-frequency components are attenuated in a first-order fashion.
- The photoelectron transport apparatus described by Equations (1)–(3) is physically a chain as depicted in Figure 6. Photoelectrons are relayed forward or backward along the chain. A photoelectron that has penetrated more deeply along the chain will need to jump more links, on a probability basis, and thus experience more time constants (or eigenvalues) of the kinetics to resurface as a DF photon. This sequential nature of the chain is not explicit in equations such as Equations (1)–(3) but can be eloquently represented in kinetic Monte Carlo simulation as shown in Guo and Tan [11]. As a result, earlier links primarily affect the high-frequency behavior while the whole links mainly contribute to the low-frequency kinetics. The spectra convergence at high frequencies shown in Figure 7 thus imply that the front links may be similar for the samples tested and the differences are chiefly in the down-stream links. To develop low-order models to represent the low-frequency behaviors, it is thus reasonable to lump or neglect the contributions by the early links as done in Guo and Tan [16] and Guo *et al.* [21].
- The observation above is further supported by the chemical stress tests performed in this work. DCMU interrupts photoelectron transport by binding to the Q_B_ sites. It alters a downstream link of the chain and thus should affect the low-frequency kinetics. Application of DCMU only affected the spectra below 0.04 Hz (Figure 5), much lower than the frequencies below which the sample species differed. Moreover, the longer the submerging time is, the more Q_B_ sites are disabled and thus the less low-frequency components there should be, which is exactly what Figure 5 shows.
- The explainable differences and similarities in the DF FT spectrum from different samples suggest that the DF FT spectrum can be used as a biosensor to evaluate plant physiology and environmental stress. The proposed technique can be a complementary method for DF signal analysis in the time domain.

## 5. Conclusions

In sum, this work shows similarities and differences in the DF spectra of different plants with or without herbicide stress. The similarities and differences are as expected and explainable. All measured spectra exhibited low-pass characteristics with high frequencies attenuated at 20 dB per decade. Earlier links in the photoelectron transport chain contribute to high-frequency characteristics, which were similar, while the whole links influence the low-frequency range, which differed. The results support the use of low-order models that focus on the states of plastoquinones. DF spectra thus may provide a potentially alternative and useful way to analyze photoelectron transport in plants and classify samples. Measurement of more plant species under different stresses and light intensity is warranted in future research.
